# Administration of ivermectin to cattle induced mortality, reduced fecundity and survivorship of *Anopheles arabiensis* in Ethiopia: an implication for expansion of vector control toolbox

**DOI:** 10.1186/s41182-023-00575-z

**Published:** 2024-01-16

**Authors:** Ephrem Damene, Fekadu Massebo

**Affiliations:** Department of Biology, Arba Minch University, Arba Minch, Ethiopia

**Keywords:** *Anopheles arabiensis*, Fecundity, Ivermectin, Mortality, Survival rate

## Abstract

**Background:**

Although many countries have shown interest in eliminating malaria, approaches that complement existing vector control interventions are needed because existing methods have been scaled up but malaria still persists. Therefore, the effect of ivermectin administration to cattle was evaluated for its effect on mortality, survivorship and mortality of laboratory reared *Anopheles arabiensis*.

**Methods:**

Three calves were randomly selected and injected with ivermectin at a therapeutic dose of 0.2 mg/kg, while the other two calves received no treatment and served as controls. Five tents were constructed for the trial. Calves were housed in tents (one per tent) and then 30 starved female *An. arabiensis* were introduced into each tent. Only fully engorged females were collected from each tent and placed in different mosquito cages to monitor their mortality, survival and fecundity. Data analysis was done using SPSS version 16.

**Results:**

During the follow-up period (until day 21), ivermectin induced significantly higher mortality when compared to controls. It resulted in an average 24-h mortality rate of 81.6% against *An. arabiensis* on the first day following treatment. 100% *An. arabiensis* that fed on ivermectin-treated calves on the first day after treatment died within four days. Egg production rate of *An. arabiensis* that fed on ivermectin-treated calves was significantly lower compared to controls (*F* = 768.7, *P* < 0.001).

**Conclusion:**

In conclusion, ivermectin induced mortality, reduced fecundity and survivorship of laboratory maintained *An. arabiensis*. Further study is recommended using a wild mosquito population. Moreover, mass ivermectin administration to domestic animals could be recommended to supplement the existing indoor based interventions.

## Introduction

Between 2000 and 2015, there has been a significant reduction in malaria incidence and mortality rates globally [1]. After 2017, the situation in Africa has deteriorated [2, 3] as there has been a resurgence in the number of cases and deaths [2, 3]. There was an increase in the number of cases and deaths in 2020, and even more cases and deaths were documented in 2022, particularly in Africa [4]. The recent increase in cases may be due to insecticide-resistant vector populations, changes in vector feeding and resting behavior, and increased antimalarial and diagnostic resistance [4]. Rapid population growth increases the number of people at risk, but the supply of insecticide-treated nets (ITNs) and indoor residual spraying (IRS) chemicals does not keep up [5]. That means there are people left behind without ITNs and IRS.

Malaria vectors mainly feed on cattle and humans outdoors are less affected by IRS and ITN [6]. In some countries, the biting hours of the malaria vectors shift to the early hours of the night before people sleep under bed nets [7–9]. In other countries, the primary malaria vectors, *An. gambiae s.s*. and *An. funestus* have been replaced by *An. arabiensis* which is more attracted to animals and bites in the early night hours [10]. *Anopheles arabiensis* has been shown to bite outdoor and in the early hours at night [7, 8], which makes it a significant contributor to the transmission of residual malaria [6]. Secondary malaria vectors respond poorly to ITN and IRS [11] and even highly anthropophilic Anopheles species tend feed on animal and outdoor in response to the indoor malaria control tools [12]. Therefore, new complementary tools are needed to fill these gaps.

Involving the domestic animals in the control of malaria is recommended in many situations [13–16] although the evidences are controversial; some claims that animals reduce malaria infection and the other claims that animal increases mosquito bites and malaria infection [17, 18]. The proximity of cattle to human houses may increase the bites of malaria vectors and the risk of malaria infection [17]. The number of animals and the way the animals deployed may influence the role of animal in malaria control. The application of topical insecticides on animal has shown a promising result against malaria [19]. Rowland and his colleagues who worked in Pakistan documented the effectiveness of application of pyrethroid insecticide on cattle against zoophilic malaria vectors and claimed up to 56% reduction of malaria incidence [19]. There are also systemic insecticides (injected into subcutaneous tissue) such as ivermectin that are effective against endo- and ectoparasites [20, 21]. Ivermectin was first introduced for commercial use as an anti-parasitic drug for the animal use in 1981 [22]. It targets the glutamate-gated chloride channels found in invertebrate post-synaptic neurons and neuromuscular junctions [22, 23]. This action hyperpolarizes the neurons and muscle fibers, leading to paralysis and insect deaths. Ivermectin's lethal potential against a variety of arthropods, including mosquitoes, has been documented [24].

We documented the potency of ivermectin on *An. arabiensis* fed on human on a number of mosquito malaria transmission capacity parameters such as longevity, fecundity and fertility in Ethiopia [16]. Additionally, the feeding behavior of malaria mosquitoes is thought to contribute to the effectiveness of ivermectin administration to cattle in the malaria vector control toolkit [24]. In this regard the main malaria vector *An. arabiensis* has shown a tendency to feed on livestock in the region [25] and therefore a focus on animals could be useful in a control program. Regardless of the wide distribution of the existing malaria vector control methods, alternative approaches to supplement the existing vector control interventions are needed as malaria yet remains. The research group sought to explore evidence for potency of ivermectin administration to domestic animals as a supplementary anti-malaria vector mosquito approach. Therefore, the effect of ivermectin administration to cattle on entomological parameters of malaria was investigated.

## Materials and methods

### Description of the experimental animals

Five local male zebu cattle with a body weight of 110–150 kg and aged 1–2 years were used (Table 1). They were treated with albendazole (5 mg/kg) two weeks before commencing the trial to avoid stress, and other health problems and avoid cross-reactions if any to ivermectin. They were housed and cared for by the owner to avoid stress and to maximize the effect of the treatment. The calves were housed individually (till the end of the experiment) in separate houses to avoid contact between treatment and control groups.

**Table 1:** Physical condition of experimental calves and ivermectin doses given to calves

Group	Code	Age in months	Mass in KG	Dose of ivermectin (0.2 mg/kg or 1 ml/50 kg)
Ivermectin treatment	Calve 1	20	125	2.5 ml
	Calve 2	19	120	2.4 ml
	Calve 3	23	140	2.8 ml
Control	Calve 1	21	130	Na
	Calve 2	22	138	Na

*Na* not applicable

### *Anopheles arabiensis* rearing

The colony of *An. arabiensis*, originally maintained for decades at the Adama Malaria Research Center, has been in the Medical Entomology and Vector Control Laboratory of Arba Minch University since 2014. The colony is susceptible to all public health insecticides. It is maintained under standard temperature (27 ± 2 °C), relative humidity (75 ± 5%) and 12 h day: 12 h night cycle. Larvae reared on plastic trays in distilled water were provided with ground Tetramin^®^ fish food (fish meal). Pupae were collected in cups, placed in 25 × 25 × 25 cm mosquito cages and emerged adults were allowed to feed on 10% sugar solution.

### Experimental design

**Fig. 1:**
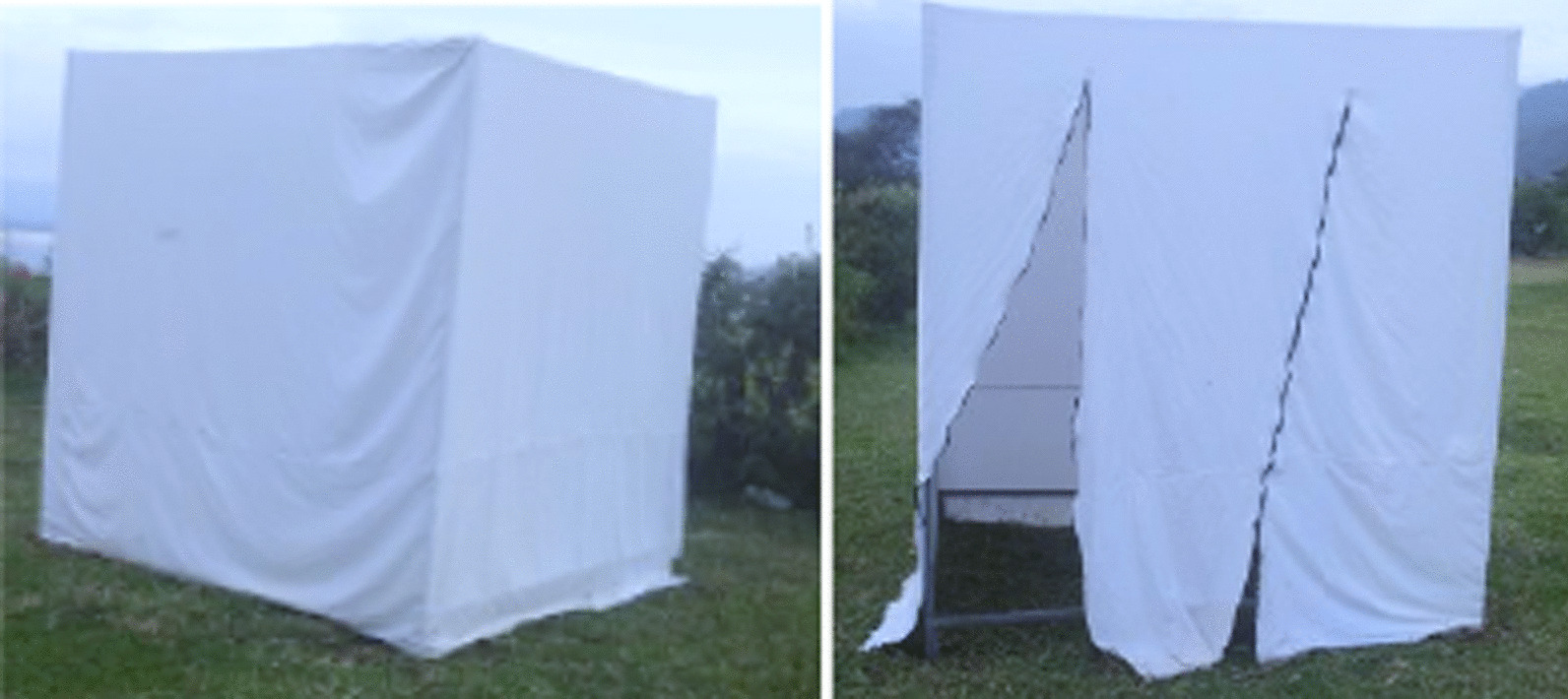
Back side (left) and front (right) of the experimental tent

Five white tents each of 2 m × 2 m × 2 m were set at Arba Minch University Abaya campus (Fig. 1). The metal frame was used as scaffolding on four sides, top and bottom. A tie was fixed inside each tent to restrict cattle movement and facilitate mosquito feeding. A white sheet of 2.5 m × 2.5 m × 2.5 m spread on the ground for easy recognition of knock down mosquitoes. We used white tents to visualize the resting mosquitoes.

Three ivermectin treated and two untreated (control) calves were assigned randomly to each tent. In each tent, 30 starved 3–5 days old female *An. arabiensis* were introduced at 18:00 and allowed to feed until 20:00 h. After that, the openings were closed with zippers to prevent mosquitoes from escaping. Feeding was done on days 1, 3, 9, 14 and 21 after ivermectin administration. The residual effect of ivermectin was assessed based on its already documented residual lifespan up to day 21 [16, 26, 27]. The same batch of laboratory-reared *An. arabiensis* was used for each feeding timeline in both control and treatment groups. Live *An. arabiensis*, including fully fed and unfed, were collected by mouth aspirator. The forceps were used to collect crushed or dead mosquitoes, which were then counted to ensure none were missing before being thrown away.

### Experimental procedures

#### Treatment of cattle with ivermectin

Ivermectin (IVOMEC D^®^) was administered subcutaneously to three randomly chosen calves at the therapeutic dose of 0.2 mg/kg of body weight, while the other two served as the control group and received no treatment. The drug was injected by a veterinarian.

#### Monitoring *An. arabiensis* mortality and survivorship

Fully engorged female *An. arabiensis* were transferred to individually labeled cages at all post-treatment feeding timeline to monitor the effect of ivermectin on morality and survival. Unfed mosquitoes were discarded after being chilled for 15 to 20 min. Three cages were set up for treatment and two for control to monitor mortality and survival. Mosquitoes were provided with a 10% sucrose solution and their mortality and survival were monitored for 12 consecutive days, taking into account the sporogonic development period of malarial mosquitoes in most cases. The number of mosquito dead in the first 24 h of feeding was recorded for mortality estimation, while the mosquitoes survived by each day for 12 consecutive days were recorded for survivorship estimation. The last post-treatment feeding experiment was performed on day 21 because of the residual lifespan of ivermectin [14, 27].

#### Monitoring *Anopheles arabiensis* fecundity

Assessment of the post-administration effect of ivermectin on *An. arabiensis* fecundity started on day 9 due to high mortality in the first few days of feeding. For fecundity estimation (the number of eggs produced), the proportion of survivors were randomly divided into two groups on day 4 after the blood meal (for filter paper egg count and for ovarian dissection to count eggs) [28]. Ovaries were extracted and dropped on distilled water to release the eggs which were then counted under a dissecting stereomicroscope. The number of eggs of a gravid female in the experiment was considered as a proxy for their fecundity. For filter paper egg count, each mosquito was transferred to a plastic container with moist filter paper to lay eggs, and then the container was covered with a nylon mesh screening. Mosquitoes were observed daily for oviposition and deposited eggs were counted under the microscope.

### Data analysis

Mean, standard deviation (SD) and standard error (SE) of mean along its 95% confidence interval (CI) were analyzed to describe the survivorship. Kaplan–Meier estimator package was used to calculate the survivorship function of a random variable in time. Survivorship curves were compared using Log Rank test. Cox regression was applied to predict the proportional hazards probability. The mean difference in the number of eggs between control and treatment groups compared using a pairwise comparison test. All analyses were carried out using statistical software, SPSS version 16.

## Results

### Description of mosquitoes exposed to feed on calves

Table 2 shows the number of *An. arabensis* exposed to calf feeding and the number collected after feeding. About 80.4% *An. arabiensis* was successfully fed to calves. Mean percentage of freshly fed *An. arabiensis* collected from treatment group was 79.5% (95% CI 75.9–83.3), and it was 81. 6% (95% CI 78.4–84.9) for control group.

**Table 2:** Description of *Anopheles arabiensis* exposed to feed on calves, freshly fed and percent freshly fed

DAI	Test	Number exposed	Number freshly fed	Percent freshly fed
1	Control	60	48	80.0
	Treatment	90	70	77.8
3	Control	60	51	85.0
	Treatment	90	72	80.0
9	Control	60	49	81.7
	Treatment	90	68	75.6
14	Control	60	50	83.3
	Treatment	90	75	83.3
21	Control	60	47	78.3
	Treatment	90	73	81.1
	Total	750	603	80.4

### Effect of ivermectin on mortality of *An. arabiensis*

**Fig. 2:**
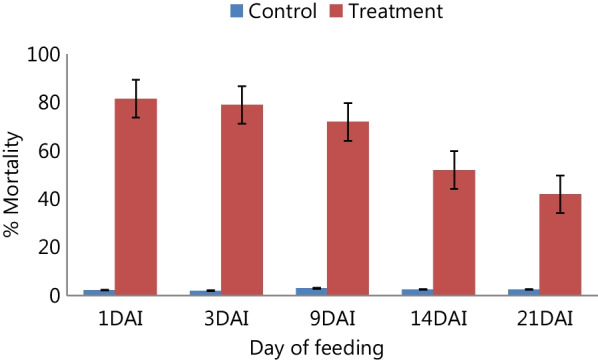
The mean *An. arabiensis* mortality during 24 h of each feeding point on ivermectin treated and control calves. *DAI* day after injection

The 24 h *An. arabiensis* mortality was significantly higher in those fed on treated calves than the controls (Table 3). Ivermectin induced significantly higher mortality until 21 days after injection (DAI) compared to controls. A higher average mortality (81.6%; 95% CI 75.5–87.7) was observed on day 1 post-treatment, which gradually decreased to 41.7% (95% CI 36.6–46.8) on day 21 post-treatment (Fig. 2).

**Table 3:** Mortality and survivorship of *An. arabiensis* that fed on ivermectin treated calves within twelve days follow-up period

DAI	Test	Mean mortality rate (95% CI)	SE	% survival reduction	*P-*value
1	Control	7.9 (3.8–12.0)	2.1	–	–
	Treatment	100	1.7	92.1	< 0.001
3	Control	11.3 (7.2–15.4)	2.1	–	–
	Treatment	100	1.7	88.7	< 0.001
9	Control	11.7 (7.8–15.6)	2.1	–	–
	Treatment	86.4 (83.1–89.7)	1.7	86.4	< 0.001
14	Control	12.1 (7.9–16.2)	2.1	–	–
	Treatment	48.3 (44.9–51.6)	1.7	74.9	< 0.001
21	Control	11.2 (7.1–15.3)	2.1	–	–
	Treatment	27 (23.7–30.3)	1.7	58.5	< 0.056

### Effect of ivermectin on the survivorship of *An. arabiensis*

The survivorship of *An. arabiensis* reduced significantly after feeding on the ivermectin treated calves compared to those fed on the controls (*F* = 110.7, *P* < 0.001). Mean survivorship of *An. arabiensis* was significantly affected by the interaction of treatment and day after injection (*F* = 15.13, *P* < 0.001). All (100%) *An. arabiensis* that fed on ivermectin-treated calves on the first day after treatment died within 4 days, whereas 7.9% of mosquitoes in the control group died 12 days after feeding. On the other hand, 27% of *An. arabiensis* fed on day 21 after treatment died within 12 days and the effect was marginally significant (*F* = 3.6, *P* = 0.056) compared to control (Table 3).

**Fig. 3:**
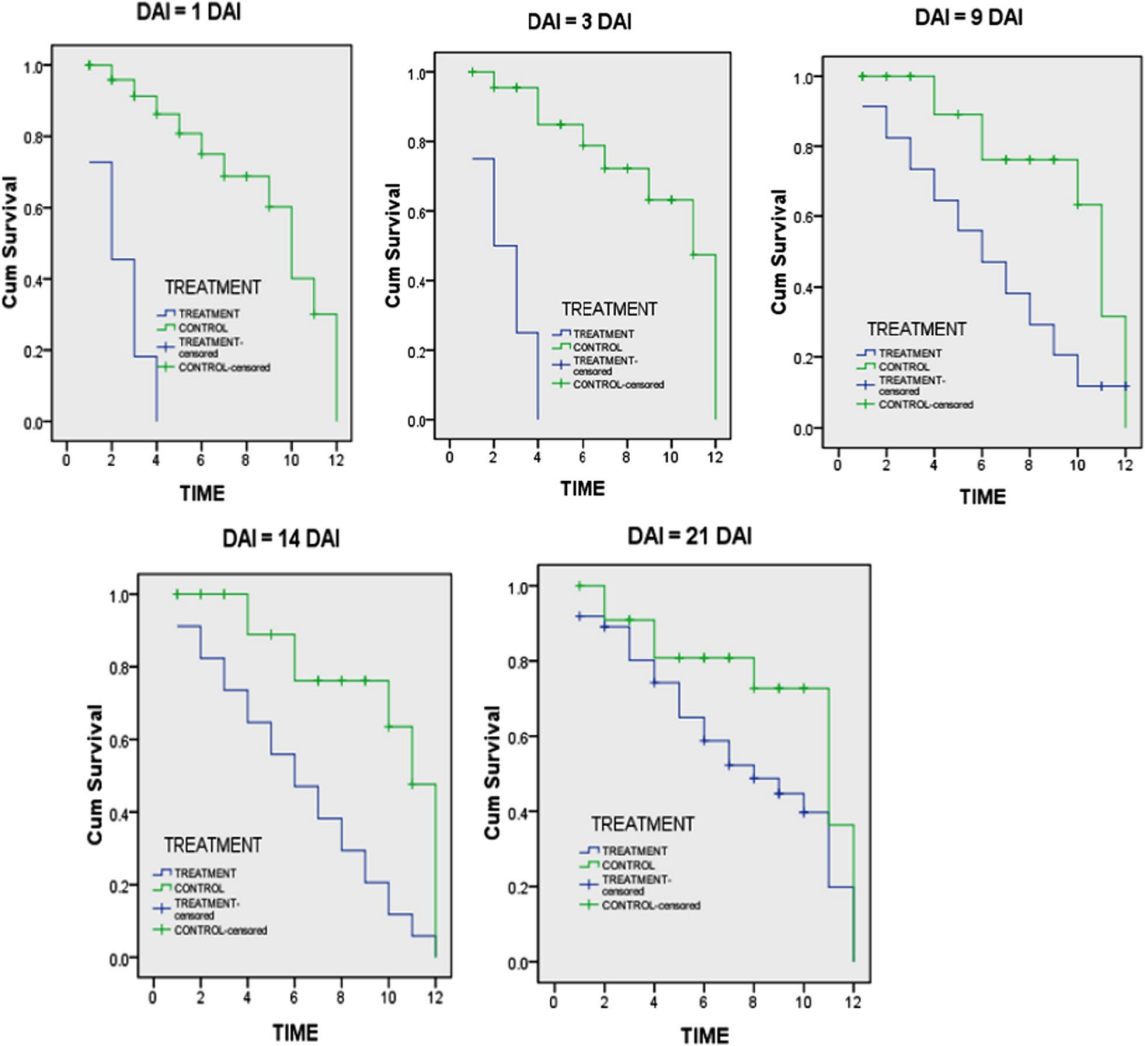
Kaplan–Meier estimates of survivorship time (in days) of *Anopheles arabiensis* feeding on calves after injection of 0.2 mg/kg of ivermectin. *Blue lines* mosquitoes fed on treated calves. *Green lines* mosquitoes fed on control calves. Censored *An. arabiensis*: those died during the following period

The Kaplan–Meier survivorship curves showed the effect of the ivermectin versus time in comparison with the control group (Fig. 3). The median survivorship time of *An. arabiensis* that fed on treated group was shorter compared to those fed on the control (Table 4). The median survivorship time was significantly short among the *An. arabiensis* that fed on ivermectin at day 1 (*F* = 27.2, *P* < 0.001), 3 (*F* = 25.9, *P* < 0.001), 9 (*F* = 9.1, *P* = 0.003), 14 (*F* = 7.9, *P* = 0.005) and 21 after treatment (*F* = 3.6, *P* = 0.006).

**Table 4:** Estimated median survivorship time (days) of *An. arabiensis* after feeding on ivermectin treated and control calves

DAI	Treatment, median days (95% CI)	Control, median days (95% CI)
1	2 (0.9–3)	10 (8.6–11)
3	2 (0.8–3)	11 (9–12)
9	6 (4–7.9)	11 (10–12)
`14	7 (5–8.7)	11 (10–12)
21	8 (4–11)	11 (8–14)

### Effect of ivermectin on fecundity of *An. arabiensis*

A total of 300 blood engorged *An. arabiensis* were used to estimate fecundity. Egg production rate of *An. arabiensis* that fed on ivermectin-treated calves was significantly lower compared to controls (*F* = 768.7, *P* < 0.001). Mean number of eggs from ovary dissection (*F* = 411.3, *P* < 0.001) and filter paper count (*F* = 435.3, *P* < 0.001) reduced significantly due to ivermectin (Tables 5, 6).

**Table 5:** Effect of ivermectin on mean number of eggs per female *An. arabiensis* from filter paper count at different points of feeding after treatment

DAI	Test	Mean no. eggs/female (95% Wald CI)	% reduction	*P-*value
9	Control	89 (82.8–95.2)	–	–
	Treatment	41 (35.9–46.1)	53.9	< 0.001
14	Control	88.5 (82.3–94.6)	–	–
	Treatment	42.7 (37.6–47.7)	51.7	< 0.001
21	Control	87 (80.8–93.2)	–	–
	Treatment	63.3 (58.3–68.4)	27.2	< 0.001

**Table 6:** Effect of ivermectin on mean number of eggs per female *An. arabiensis* by dissecting ovaries at different points of feeding after treatment

DAI	Test	Mean no. eggs/female (95% Wald CI)	% reduction	*P-*value
9	Control	90 (85–95)	–	–
	Treatment	28.3 (24.2–32.5)	61.7	< 0.001
14	Control	82.5 (77.5–87.5)	–	–
	Treatment	34.7 (30.5–38.5)	57.9	< 0.001
21	Control	88.5 (83.5–93.5)	–	–
	Treatment	62.5 (58.5–67.5)	29.4	< 0.001

## Discussion

This study evaluated the effect of cattle treatment with ivermectin on mortality, survivorship and fecundity of *An. arabiensis*. Ivermectin induced mortality, reduced survivorship and fecundity of *An. arabiensis* for a minimum of 21 day after injection. The proportion of surviving and their median survivorship times of *An. arabiensis* were reduced in those feeding on ivermectin treated calves compared to control calves. However, the effects gradually declined after two weeks of ivermectin administration and may require repeated injections of animals during long transmission seasons. Similarly, Lyimo et al. [28] reported five-fold higher mortality of mosquitoes fed on ivermectin treated cattle relative to control in the first week. Other studies have reported higher mortality in *An. arabiensis* that were fed on cattle treated with ivermectin [26, 28]. This reveal that greater number of mosquito can die after feeding on ivermectin treated calves. Therefore, it may be important for fast reduction of vector population and hence, may reduce human-vector contact.

Feeding calves treated with ivermectin reduced the proportion of survivor *An. arabiensis* and their mean survival time compared to controls. The low survivorship of malaria vectors due to ivermectin implies the possibility of dying before the next blood meal and become infectious. Moreover, Pooda et al. [29] reported 75% of mosquito mortality before able to become infectious after feeding on treated cattle. Shorter life of those mosquitoes fed on ivermectin treated cattle has also been documented by Lyimo et al. [28]. Strategies which target the longevity of mosquitoes are obviously important to interrupt malaria transmission because the development of parasites require extrinsic incubation period. As ivermectin targets the adult mosquitoes and reduce mosquito survivorship which leads to a shift in the population structure to younger mosquitoes and hence a lower proportion of infectious mosquitoes. This provides evidence that treating cattle with ivermectin could impact the longevity of malaria vectors and reduce the number of infectious mosquitoes.

The present study also revealed that ivermectin could reduce egg production rate of *An. arabiensis* at least for 21 days. Some studies also showed similar findings [14, 17, 29]. Treatment of humans with ivermectin also induced the similar effect against malaria vectors [5, 16]. This suggests that feeding ivermectin-treated cattle can decrease the density of *An. arabiensis* in the successive generations that have a direct impact on the transmission of malaria. Today, integrating interventions to maximize benefit is strongly recommended. Ivermectin is widely used to control endo- and ectoparasites in animals and to treat filarial nematode parasites in humans [27]. It has a different mode of action than the insecticides used to control malaria vectors, potentially making it even useful for insecticide-resistant vector populations [20, 21]. Hence, ivermectin based intervention could be deployed in conjunction with other WHO-recommended malaria control measures and defining the ideal context for ivermectin based tool could be practically voluble to control the zoophilic malaria vector in the region [25].

This study has strengths and limitations. This study provided further evidence of the effectiveness of ivermectin administration to cattle as an alternative malaria vector control agent in Ethiopia, where there are limited studies documenting results similar to the present study. The current study adds credence to the extrapolation of the animal-based malaria vector strategy to the wider ivermectin-using community. Integrating the veterinary and public sectors can be beneficial for policymakers as it can help in maximizing resource utilization. In areas where malaria is prevalent, animal endo- and ectoparasites are also common, leading to health and economic problems in communities. Animal-based malaria control programs can have a dual effect by addressing both economic and health issues [29, 30]. It is important to note that using laboratory-reared mosquitoes may not provide accurate information about the wild population. Additionally, the study did not assess the impact of ivermectin on transmission-blocking or parasite development in mosquitoes. Finally, it would be beneficial to evaluate the effect of mass ivermectin administration to domestic animals at the community level to determine whether it can supplement existing indoor-based interventions.

## Conclusion and recommendations

Ivermectin induced mortality, reduced survivorship and fecundity of *An. arabiensis* for a minimum of 21 day after therapy. Proportion of survivors and the average survival time of *An. arabiensis* that fed on ivermectin treated calves was low compared to controls. These results notify that ivermectin treated calves may suppress *An. arabiensis* population and malaria transmission, and it could be integrated with the IRS and ITNs to control the zoophilic malaria vectors.

## Data Availability

The data supporting the conclusions of this article are included within the manuscript. The data will be available up on request.

## References

[1] Bhatt S, Weiss DJ, Cameron E, Bisanzio D, Mappin B, Dalrymple U (2015). The effect of malaria control on *Plasmodium falciparum* in Africa between 2000 and 2015. Nature.

[2] WHO (2021). World malaria report 2021.

[3] Jagannathan P, Kakuru A (2022). Malaria in 2022: increasing challenges, cautious optimism. Nat Commun.

[4] WHO. World Malaria Report. Geneva: World Health Organization; 2023.

[5] Russell TL, Beebe NW, Cooper RD, Lobo NF, Burkot TR (2013). Successful malaria elimination strategies require interventions that target changing vector behaviours. Malar J.

[6] Cohen AJM, Okumu F, Moonen B (2022). The malaria fight’s diminishing gains and growing challenges. Sci Transl Med..

[7] Russell TL, Govella NJ, Azizi S, Drakeley CJ, Kachur SP, Killeen GF (2011). Increased proportions of outdoor feeding among residual malaria vector populations following increased use of insecticide-treated nets in rural Tanzania. Malar J.

[8] Kenea O, Balkew M, Tekie H, Gebre-Michael T, Deressa W, Loha E (2016). Human-biting activities of Anopheles species in south-central Ethiopia. Parasites Vectors.

[9] Sherrard-Smith E, Skarp JE, Beale AD, Fornadel C, Norris LC, Moore SJ (2019). Mosquito feeding behavior and how it influences residual malaria transmission across Africa. Proc Natl Acad Sci USA.

[10] Derua YA, Alifrangis M, Hosea KM, Meyrowitsch DW, Magesa SM, Pedersen EM (2012). Change in composition of the *Anopheles gambiae* complex and its possible implications for the transmission of malaria and lymphatic filariasis in north-eastern Tanzania. Malar J.

[11] Mwangangi JM, Mbogo CM, Orindi BO, Muturi EJ, Midega JT, Nzovu J (2013). Shifts in malaria vector species composition and transmission dynamics along the Kenyan coast over the past 20 years. Malar J.

[12] Service MW (1991). Agricultural development and arthropod-borne diseases: a review. Rev Saude Publica.

[13] Chaccour C (2021). Veterinary endectocides for malaria control and elimination: prospects and challenges. Philos Trans R Soc Lond B Biol Sci.

[14] Khaligh FG, Jafari A, Silivanova E, Levchenko M, Rahimi B, Gholizadeh S (2021). Endectocides as a complementary intervention in the malaria control program: a systematic review. Syst Rev.

[15] Slater HC, Foy BD, Kobylinski K, Chaccour C, Watson OJ, Hellewell J (2020). Ivermectin as a novel complementary malaria control tool to reduce incidence and prevalence: a modelling study. Lancet Infect Dis.

[16] Mekuriaw W, Balkew M, Messenger LA, Yewhalaw D, Woyessa A, Massebo F (2019). The effect of ivermectin^®^ on fertility, fecundity and mortality of *Anopheles arabiensis* fed on treated men in Ethiopia. Malar J.

[17] Donnelly B, Berrang-Ford L, Ross NA, Michel P (2015). A systematic, realist review of zooprophylaxis for malaria control. Malar J.

[18] Bouma M, Rowland M, Report WM (1995). Failure of passive zooprophylaxis: cattle ownership in Pakistan is associated with a higher prevalence of malaria. Trans R Soc Trop Med Hyg.

[19] Rowland M, Durrani N, Kenward M, Mohammed N, Urahman H, Hewitt S (2001). Control of malaria in Pakistan by applying deltamethrin insecticide to cattle: a community-randomised trial. Lancet.

[20] Ruiz-Castillo P, Rist C, Rabinovich R, Chaccour C (2022). Insecticide-treated livestock: a potential One Health approach to malaria control in Africa. Trends Parasitol.

[21] Kositz C, Bradley J, Hutchins H, Last A, D’Alessandro U, Marks M (2021). Broadening the range of use cases for ivermectin—a review of the evidence. Trans R Soc Trop Med Hyg.

[22] Campbell WC, Fisher MH, Stapley EO, Albers-Schonberg G, Jacob TA (1983). Ivermectin: a potent new antiparasitic agent. Science.

[23] Meyers JI, Gray M, Kuklinski W, Johnson LB, Snow CD, Black WC (1991). Characterization of the target of ivermectin, the glutamate-gated chloride channel, from *Anopheles gambiae*. Rev Saude Publica.

[24] Billingsley P, Binka F, Chaccour C, Foy BD, Gold S, Gonzalez-Silva M (2020). A roadmap for the development of ivermectin as a complementary malaria vector control tool. Am J Trop Med Hyg.

[25] Massebo F, Balkew M, Gebre-Michael T, Lindtjørn B (2015). Zoophagic behaviour of anopheline mosquitoes in southwest Ethiopia: opportunity for malaria vector control. Parasites Vectors.

[26] Chaccour C, Lines J, Whitty CJM (2010). Effect of ivermectin on *Anopheles gambiae* mosquitoes fed on humans: the potential of oral insecticides in malaria control. J Infect Dis.

[27] Chaccour CJ, Kobylinski KC, Bassat Q, Bousema T, Drakeley C, Alonso P (2013). Ivermectin to reduce malaria transmission: a research agenda for a promising new tool for elimination. Malar J.

[28] Lyimo IN, Kessy ST, Mbina KF, Daraja AA, Mnyone LL (2017). Ivermectin-treated cattle reduces blood digestion, egg production and survival of a free-living population of *Anopheles arabiensis* under semi-field condition in south-eastern Tanzania. Malar J.

[29] Pooda HS, Rayaisse JB, Hien DFDS, Lefèvre T, Yerbanga SR, Bengaly Z (2015). Administration of ivermectin to peridomestic cattle: a promising approach to target the residual transmission of human malaria. Malar J.

[30] Omura S, Crump A (2014). Ivermectin: panacea for resource-poor communities?. Trends Parasitol.

